# Bilateral vestibulopathy – insight in impact on quality of life and economic burden

**DOI:** 10.1007/s00405-025-09692-3

**Published:** 2025-10-14

**Authors:** F M P Lucieer, S. Paredis, A. Perez-Fornos, N. Guinand, V. Van Rompaey, H. Kingma, M. Joore, R. van de Berg

**Affiliations:** 1 Department of Otorhinolaryngology and Head and Neck Surgery, Division of Balance Disorders, School for Mental Health and Neuroscience, Maastricht University Medical Center+, Maastricht, Netherlands; 2 Service of Otorhinolaryngology Head and Neck Surgery, Department of Clinical Neurosciences, Geneva University Hospitals, Geneva, Switzerland; 3 Department of Otorhinolaryngology and Head and Neck Surgery, Faculty of Medicine and Health Sciences, Antwerp University Hospital, University of Antwerp, Antwerp, Belgium; 4 Department of Otorhinolaryngology and Head and Neck Surgery, Division of Balance Disorders, faculty of Medicine, Aalborg University Hospital, Aalborg, Denmark; 5 Department of Clinical Epidemiology and Medical Technology Assessment (KEMTA), Care and Public Health Research Institute (CAPHRI, Faculty of Health, Medicine and Life Sciences of Maastricht University (FHML), Maastricht University Medical Centre+, Maastricht, The Netherlands

**Keywords:** Bilateral vestibulopathy, Vestibular, Quality of life, Economic burden

## Abstract

**Objective:**

To investigate the impact of bilateral vestibulopathy (BV) on quality of life, capabilities and costs. Furthermore, to estimate the potential headroom of a vestibulocochlear implant treatment trajectory, and comparing it to the anticipated costs.

**Methods:**

Patients meeting the Bárány Society diagnostic criteria for BV were included. Data on quality of life (EQ-5D-5L) and capabilities (ICECAP-A) were obtained, along with BV related health care costs, and patient and family costs (last year and total disease duration) through a survey and interview. Productivity loss was measured using PRODISQ and Friction Cost Method. The one year and lifetime headroom were calculated. Experts were interviewed about anticipated health care consumption for a future treatment trajectory.

**Results:**

Fifty patients participated. The mean utility EQ-5D-5L score was 0.680, and the ICECAP-A score was 0.839. Mean total costs in the last year were €5,388 and €30,708 for the total disease duration. The one-year headroom was €12,261; and €306,489 for lifetime. The future treatment trajectory costs were €32,605 for the first year (excluding the implant and processor).

**Conclusion:**

BV patients have significantly lower health related quality of life compared to the general population. Combined with their health care consumption and other costs, this provides sufficient headroom for a potentially cost-effective vestibulocochlear implant treatment trajectory.

**Supplementary Information:**

The online version contains supplementary material available at 10.1007/s00405-025-09692-3.

## Introduction

Bilateral vestibulopathy (BV) is a chronic disorder in which the vestibular function is bilaterally absent or severely reduced [1, 2]. BV results in a variety of symptoms [1]. Symptoms for diagnosis include unsteadiness when walking or standing (worsening in darkness and on uneven ground), and oscillopsia [3]. BV has a negative impact on quality of life [4, 5]. Furthermore, it was demonstrated that vestibular disorders have a negative socio-economic impact [5, 6].

Currently, as no treatment is available to restore vestibular function, vestibular rehabilitation therapy is the recommended treatment option [7]. Vestibular rehabilitation therapy consists of education, an exercise-based treatment program, and home exercises [7]. Unfortunately, rehabilitation is not sufficient in the majority of patients. Therefore, the concept of a vestibulocochlear implant was developed, to restore vestibular function [8, 9]. The vestibulocochlear implant, analogous to the cochlear implant, captures motion and processes it into electrical signals. These electrical signals are then transferred to the vestibular nerves. The vestibular nerves are stimulated, with the aim to improve vestibular function. The first vestibulocochlear implant trials demonstrated (partial) restoration of the vestibulo-ocular reflex and vestibulo-colic reflex, and improvement of controlled postural responses. Additionally, functional benefits (normalization of dynamic visual acuity), and improvement of quality of life were demonstrated [10–15]. Therefore, the vestibulocochlear implant is a promising treatment option in the near future. However, the costs of this treatment option are likely to be substantial.

During the development of a new medical device, it is important to investigate the potential impact on quality of life and the cost-effectiveness of the device. For this, insight in the generic health-related quality of life, health care consumption, patient and family expenses, and productivity of patients receiving current care, is crucial. Regarding the vestibulocochlear implant, this information is lacking for the European context. Furthermore, early insight into the possible costs related to the potential gain in quality of life, is helpful to assess to financial sustainability of this treatment option.

The objective of this study was to obtain insight into the impact on quality of life, capabilities, and cost consequences of BV. Furthermore, this study aimed to estimate the potential headroom of a patient treatment trajectory with vestibulocochlear implants, and to compare it to the anticipated costs of this trajectory.

## Methods

### Patient population

Patients previously diagnosed with BV according to the Bárány Society diagnostic criteria at Maastricht University Medical Center+, were contacted [3]. This included patients with reported unsteadiness and/or oscillopsia during walking or head movements, and a reduced bithermal caloric response (sum of bithermal mean peak slow phase eye velocity on each side < 6°/sec) and/or a reduced vestibular-ocular-reflex (VOR) gain (< 0.6 bilaterally measured by the horizontal video head impulse test (VHIT), and/or < 0.1 measured by torsion swing test). Subjects who were not able or willing to talk about one of the investigated topics (e.g. psychological complaints, health care utilization), who were not able to stop medication against anxiety or depression for at least one week due to the vestibulo-suppressive effect, or not willing to undergo one of the detailed physical, audiometric or vestibular examinations, were excluded from participation in this study. During a one day visit to Maastricht University Medical Center+, all subjects were interviewed and underwent vestibular testing by the same examiner (FL) [1]. Surveys and instruments were completed before the one-day visit.

### Vestibular testing

The caloric test was performed in a completely dark room. In supine position, warm (44 °C) and cold (30 °C) water irrigations of at least 250 milliliters were administered for 30 s. Eye movements were recorded with electronystagmography (KingsLab 1.8.1, Maastricht University, Maastricht, the Netherlands). The video Head Impulse Test was performed using the Otometrics system (Otometrics, Taastrup, Denmark). The testing methods were previously described [16]. In summary, the patient was sitting in an immobile chair and was instructed to look at a fixed target on the wall at 1.5 m. Unpredictable head impulses with a velocity of > 150°/s and low amplitude (± 20°) were applied in the plane of both horizontal semicircular canals. At least seven impulses were applied in each direction.

The torsion swing test was performed using a rotatory chair (Ekida GmbH, Buggingen, Germany) with sinusoidal rotations at a frequency of 0.1 Hz and a peak velocity of 60°/s in a complete dark room. Eye movements were recorded with electronystagmography (KingsLab 1.8.1, Maastricht University, Maastricht, The Netherlands).

### Quality of life and capability well-being

The EQ-5D-5L was used to evaluate health-related quality of life [17]. The Dutch version of the ICEpop Capability Measure for Adults (ICECAP-A) was used to asses capability well-being [18–20]. The ICECAP-A assesses five capabilities that are important to one’s well-being, and to do the things they value in life. Both instruments were sent to be self-completed before the one-day research visit.

### Costs of current care

The costs of current care related to BV were collected for the last year of disease (defined as the last year up to the one-day research visit) and for the self-reported total duration of the disease. Health care costs, patient and family costs (including out of pocket costs, costs of informal care and transportation), and productivity loss, were taken into consideration. GP contacts and consumption related to falls were, because of anticipated recall problems, only asked for the previous year. Physical therapy, rehabilitation, alternative medicine, other treatments, medical devices, and changes at home were only asked for the total duration of the disease, as these might not have occurred during the last year of disease. All other questions were asked for the total duration of the disease as well as for the last year of disease.

For health care, and patient and family costs, a survey with open questions was used (supplementary materials 1). Subsequently an interview was conducted, to collect additional information and clarifications on the responses to the questionnaire. All costs were converted to the 2021 price level using consumer price index figures from the Dutch Central Bureau of Statistics [21].

The transportation costs per month (taxi, public transportation, family/friends driving) were collected. In case the distance was unknown, the average distance a person drives or uses public transportation per day of the Statistics Netherlands (Centraal Bureau voor Statistiek) in 2023 was used (post-COVID19 pandemic; based on reports of the Dutch Central Bureau of Statistics [22]).

The productivity loss was measured using the PRODISQ and valued using the friction cost method according to the Dutch guidelines [21]. The principle of the friction cost method is based on the assumption that replacement takes place in the labor market. The maximum leave of absence was 120 workdays and the average hour rate €36.25 in 2021.

### The anticipated first-year health care costs of a hypothetical treatment trajectory involving a vestibulocochlear implant

A survey about a hypothetical vestibulocochlear implant treatment trajectory was sent to 14 health care professionals (see Supplementary Materials 2). This included questions about which health care professionals they recommended, the amount of visits, and which (vestibular) examinations should be performed at what point in time. Since a cochlear implant trajectory already exists, and the vestibular function is an additional part, the health care consumption and costs of the cochlear implant treatment trajectory were used as a basis (based on the public lists of costs of the Maastricht UMC+ [23]). The vestibulocochlear implant and processor will replace the cochlear implant, therefore, the costs of the cochlear implant were not included. The vestibulocochlear implant and processor are research devices, the costs are still unknown and were not taken into account in this possible future treatment trajectory.

### Analysis

Descriptive statistics were used to describe the patient characteristics.

The EQ-5D-5L utility value was calculated using the Dutch tariff [21, 24], which reflects health state valuations obtained from a representative sample of the Dutch population. The calculated mean utility was compared to the age-matched utility of Dutch citizen of 60 years old (reference values: 0.839 [24, 25]). The Dutch version of the ICECAP-A was used to asses capability well-being [18–20]. The tariff for the general Dutch population was used with a reference value of 0.88 [19].

The costs of current care were divided into health care costs, patient and family costs, and productivity losses. For each patient, the total costs of current care were calculated for the last year of disease, the total duration of the disease, and an average per annum over the reported disease duration. Using bootstrapping (5000 samples) the mean costs and 95% confidence intervals were determined with IBM SPSS statistics version 28.0.1.0.

A headroom analysis was performed to quantify the maximum cost at which an intervention remains cost-effective [26]. The first step in the calculation was to determine the effectiveness gap. The effectiveness gap represents the potential QALY gain, if the patient population’s health were fully restored to the level of the general population. The EQ-5D-5L utility of each individual patient was compared to the score from the Dutch age-matched reference group [24]. The difference (the effectiveness gap) was calculated and tested using the paired T-test with IBM SPSS statistics version 28.0.1.0 [24, 25, 27]. Subsequently, the QALY threshold for the total study population was determined using the iMTA disease burden calculator [28]. The mean one-year headroom was calculated by multiplying for each patient the mean effectiveness gap with the threshold and adding the average BV related costs per annum over the reported disease duration. The latter was chosen because it was more representative compared to the mean of the last year of disease. Next, this headroom was averaged over all patients. A lifetime headroom was approximated, assuming that the average costs per year patients reported over their total duration of disease would be representative for their remaining life expectancy. So, the lifetime headroom was calculated by bootstrapping the mean of the individual one-year headroom multiplied by the individual life expectancy. The individual life expectancy was calculated based on age and gender according to the Dutch central statistical office [29]. A scenario analysis with the mean costs of the last year was also performed.

Additionally, the costs of a one-year hypothetical trajectory with a vestibulocochlear implant were calculated. The costs of this hypothetical trajectory were compared to the headroom, in order to explore the potential cost-effectiveness of this novel treatment option.

### Ethical considerations

This study was in accordance with the legislation and ethical standards on human experimentation in the Netherlands and in accordance with the Declaration of Helsinki (amended version 2013). Approval was obtained from the ethical committee of Maastricht University Medical Center+ (NL52768.068.15/METC). All procedures were performed at the Maastricht University Medical Center+. All subjects provided written informed consent.

## Results

### Patient characteristics

Seventy-six patients previously diagnosed with BV were approached for participation in this study. Fifty BV-patients (mean age 60 years (range 21–79 years), 50% female) were included and completed the questionnaires and interview in the period 2016–2018. The etiologies included ototoxicity (*n* = 11), infection (meningitis *n* = 3, Lyme’s disease *n* = 1, herpes infection *n* = 1, neuritis *n* = 1), hereditary (DFNA9 gene mutation *n* = 3, other *n* = 2), congenital *n* = 1, Menière’s disease (*n* = 3), auto-immune (*n* = 2), renal failure (*n* = 1), and idiopathic (*n* = 21, of which 9 reported a migraine history [30]). Forty-five BV-patients (90%) had a bilaterally reduced caloric response, thirty-nine patients (78%) had a bilateral VOR gain < 0.6 measured with the video head impulse test, and thirty patients (60%) had a VOR gain of < 0.1 on the torsion swing test. Twenty-six BV-patients (52%) met all three of the vestibular testing inclusion criteria [3]. The mean duration of disease was 11 years (SD = 13 years), median 6 years, and range 0.5–64 years. Duration of disease was < 2 years in 9 patients, 2 to 3 years in 8 patients, and > 3 years in 33 patients. These patient characteristics were previously described [1]. Twenty-six patients (out of 76) were excluded. The reasons for exclusion were: not meeting the newest Bárány Society diagnostic criteria (*n* = 4), inability to stop anxiety or depression medication (*n* = 3), travel distance and/or the demanding nature of a full test day (*n* = 7), unwillingness to participate (*n* = 5), presence of other severe illnesses (*n* = 3), and no response after receiving the information (*n* = 4).

### Quality of life

Table 1 and supplementary materials 3 present results of the EQ-5D-5L and ICECAP-A. For all dimensions, except the self-care dimension of the EQ-5D-5L, the majority of patients reported problems. Regarding the EQ-5D-5L, most problems were reported for the dimension ‘mobility’. For the ICECAP-A, most problems were reported for the dimension ‘achievement’. A minority of the patients reported moderate to severe problems (EQ-5D-5L levels 4 and 5 and ICECAP-A levels 1 and 2). The mean EQ-5D-5L VAS was 66 (SD = 19), and the mean utility was 0.680 (SD = 0.151). The mean utility for men was 0.686 (SD = 0.162), and for women 0.673 (SD = 0.141). The mean ICECAP-A capability value was 0.839 (SD = 0.144). This was 0.785 (SD = 0.228) for men and 0.820 (SD = 0.220) for women.

**Table 1:** EQ-5D-5L and ICECAP-A results per dimension

**EQ-5D-5L**	**Mobility** ***N (%)***	**Self-care** ***N (%)***	**Usual activities** ***N (%)***	**Pain and discomfort** ***N (%)***	**Anxiety and depression** ***N (%)***
1 (No problems)	4 (8%)	31 (62%)	6 (12%)	14 (28%)	22 (44%)
2 (slight problems)	9 (18%)	14 (28%)	20 (40%)	12 (24%)	17 (34%)
3 (Moderate problems)	24 (48%)	3 (6%)	16 (32%)	19 (38%)	10 (20%)
4 (Severe problems)	13 (26%)	2 (4%)	7 (14%)	5 (10%)	1 (2%)
5 (Extreme problems/Unable to do)	0 (0%)	0 (0%)	1 (2%)	0 (0%)	0 (0%)
Total	50 (100%)	50 (100%)	50 (100%)	50 (100%)	50 (100%)
Number reported some problems	46 (92%)	19 (38%)	44 (88%)	36 (72%)	28 (56%)
**ICECAP-A**	**Stability** ***N (%)***	**Attachment** ***N (%)***	**Autonomy** ***N (%)***	**Achievement** ***N (%)***	**Enjoyment** ***N (%)***
1 (no capability)	0 (0%)	0 (0%)	0 (0%)	4 (8%)	0 (0%)
2	9 (18%)	6 (12%)	12 (24%)	20 (40%)	13 (26%)
3	30 (60%)	20 (40%)	30 (60%)	21 (42%)	23 (46%)
4 (Full capability)	11 (22%)	24 (48%)	8 (16%)	5 (10%)	14 (28%)
Total	50 (100%)	50 (100%)	50 (100%)	50 (100%)	50 (100%)
Number reported some problems	39 (78%)	26 (52%)	42 (84%)	45 (90%)	36 (72%)

### Costs of current care

#### Consultations

**Fig. 1:**
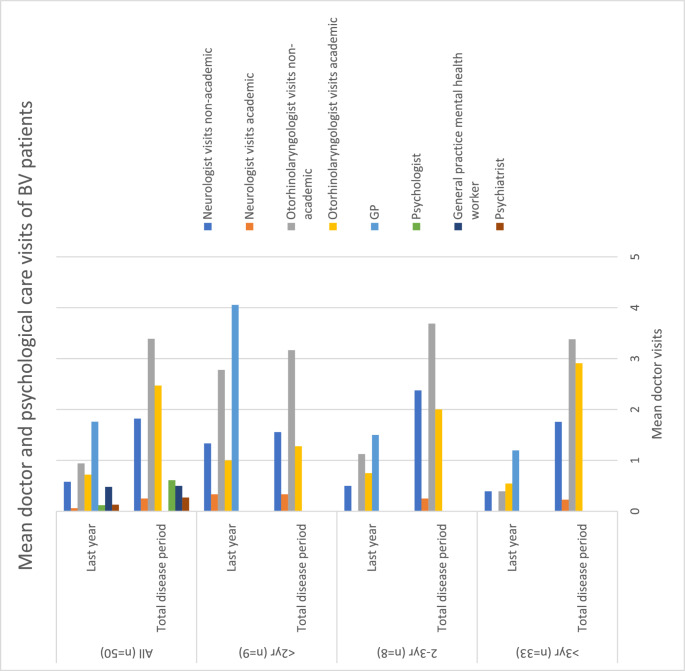
Mean doctor and psychological care visits of 50 BV patients in the last year of the disease, and during the total duration of the disease. The visits to specialists are divided into non-academic and academic hospital visits. The results of the total group are presented, as well as subgroups divided by duration of disease (< 2 years; 2–3 years; >3 years). For each subgroup, the mean visits of the last year are illustrated (last year), as well as an estimate of the mean visits during the total disease period (total)

The patients consulted general practitioners, neurologists, otorhinolaryngologists, psychiatrists, psychologists and the general practice mental health worker, with complaints related to BV. An overview of the amount of consultations is presented in Fig. 1. The amount of doctor visits decreased with the duration of disease. However, patients who had BV for over 3 years, still consulted doctors for BV related problems, mainly the general practitioner (1.76 times, SD = 4.2, 20 patients). Over the total disease duration, it was observed that the non-academic specialists were more often visited compared to their academic counterparts.

#### Costs

The total bootstrap mean costs per patient was €30,708 (95% CI 23,176–38,914) for the total disease duration. The mean costs in the last year of disease was €5,388 (95% CI 2,407–10,730). The mean annual costs, taking into account the reported disease duration, amounted to €8,863 (95% CI 5,614–12,785). A complete overview of the costs is provided in the supplementary materials 4.

Furthermore, it was found that 22 out of 33 patients of the working-age population (67%), were (partially) not able to work. Fifteen patients (45%) were on disability leave.

### Headroom analysis and costs of hypothetical future patient trajectory for vestibulocochlear implantation

The mean effectiveness gap compared to the age-matched Dutch population was 0.170 (*p* < 0.001, 95% CI 0.130–0.211). Hence, the one-year headroom amounted to €12,261 (95% CI €8,602 – €16,457). The remaining average life span of this population was 25 years [29]. The lifetime headroom amounted to €306,489 (95% CI €203,910 - €433,370). A scenario analysis with the mean costs of the last year of disease showed a one-year headroom of €8,785 (95% CI €5,517 – €14,191) and a lifetime headroom of €214,758 (95% CI €132,622 - €345,878).

The survey of a hypothetical future patient treatment trajectory for vestibulocochlear implant was returned by two otorhinolaryngologists, six audiologists, one psychologist, and one social worker. All were experienced in vestibular medicine. One otorhinolaryngologist and five audiologists completed all questions in the survey. The other experts only felt comfortable completing the topics of their own expertise. The experts recommended additional vestibular testing pre-implantation, in addition to the tests already included in the cochlear trajectory. Post-implantation, additional physical therapy sessions and psychological guidance were recommended. The total costs of the first year of the vestibulocochlear implant treatment trajectory without the implant and processor, amounted to €32,605 (details in supplementary materials 5).

## Discussion

This study comprised an assessment of quality of life and economic burden of bilateral vestibulopathy in Europe. It demonstrated a significant loss of quality of life resulting from BV (loss of 0.170 QALY). Besides, all patients experienced loss of capability in four of the five dimensions. Furthermore, the mean costs of BV were estimated at €5,388 for the last year of disease and €30,708 for the total duration of disease. The mean annual costs over the reported disease duration were €8,863. Two third of BV patients in the working-age group were (partially) not able to work. A hypothetical vestibulocochlear implant treatment trajectory might cost €32,608 for the first year (excluding the vestibulocochlear implant and processor). The headroom analysis showed that the costs of a potential treatment should be less than €12,261 per patient per year, and €306,489 for the duration of their life expectancy.

A significant loss of quality of life of BV patients was found in this study, which is congruent with previous studies [4–6]. These studies were often smaller, and used different instruments (HUI3 and/or DHI). Consequently, a reliable comparison between studies was not possible. However, it should be noted that a severe disability in BV patients was found [4–6]. This study showed an average loss of 0.170 QALY based on the EQ-5D-5L. This suggests potential for improvement, when using a possible new treatment modality. Cochlear implant studies showed a health utility value increase of 0.12 QALY (as measured with HUI3) [31]. It could be hypothesized that the vestibulocochlear implant will have a similar effect.

All patients experienced loss of capability in four out of five well-being dimensions of the ICECAP-A. Compared to the loss of quality of life, this number (0.839) was not as low as expected. However, the EQ-5D-5L and ICECAP-A evaluate different aspects of quality of life, which could explain the different results. Therefore, these instruments could be considered complimentary for future research.

In previous studies from the United States, a total dizziness-related annual economic burden of $13,019 was described for American BV patients [5]. Additionally, the average individual lifetime economic burden for vestibular disorders in American adults ranged from $38,363 to $91,241, depending on the age group [6]. After sensitivity analyses, a societal lifetime economic burden of vestibular loss was estimated to range from $113 to 404 billion [6]. However, the Dutch healthcare system is less expensive than the American healthcare system [32, 33]. For example, in the Netherlands, a referral from a general practitioner is required to visit a medical specialist in the hospital, which helps keep the number of hospital visits to a minimum. Therefore, the amounts reported in American studies cannot be directly compared to the results of this study. Nevertheless, these studies do confirm the significant economic burden caused by BV.

In this study, the majority of BV patients in the working-age group were affected in their productivity. This is congruent with previous literature, in which almost three-quarters of BV patients were on disability [34] and showed a significantly higher workplace absenteeism [5]. This results in a societal economic burden, which could be (partially) prevented with a possible treatment.

The headroom analysis indicated that a potential treatment should have a cost of less than €12,261 per patient per year and €306,489 for the remainder of their life expectancy. The costs of a hypothetical vestibulocochlear implant trajectory were calculated only for the first year including the implantation surgery, and estimated to be €32,605. The costs were quite similar to the costs of the cochlear implant trajectory in the Netherlands [23, 35]. However, due to the unknown costs of the implant and processor, these could not be included in this studies calculations. In the future, once the costs of the vestibulocochlear implant are determined, further research should be conducted to gain a more precise understanding of the exact costs associated with the vestibulocochlear implant trajectory and the follow-up [36]. The costs of the first year of the vestibulocochlear trajectory are higher than the one-year headroom. However, it is expected that the annual costs of the trajectory will be less after the first the year. After all, follow-up is expected to include only annual (or biennial) visits and processor replacement every five years, similar to the cochlear implant trajectory [35, 37]. The total costs of vestibulocochlear implantation over the lifespan of patients with BV are likely to be well below the headroom.

### Limitations

The costs were estimated based on self-report and may have suffered from recall bias. Most likely this resulted in an underestimation of the total costs and falls related to BV. After all, patients were asked to remember visits, diagnostic tests and previous treatments since the onset of their symptoms, while BV was present in most patients for more than 3 years. Furthermore, patient and family costs were also likely underestimated in this study. Patients often mentioned that their partner took on many household chores (e.g. cleaning, grocery shopping, gardening), as well as driving them around. Since patients considered this to be normal, they often did not recognize it as caregiver support.

## Conclusion

BV patients have significantly lower health related quality of life compared to the general population. Combined with their health care consumption and other costs, this provides sufficient headroom for a potentially cost-effective vestibulocochlear implant treatment trajectory.

## Supplementary Information

Below is the link to the electronic supplementary material.

Supplementary Material 1 (PDF 507 KB)
