# Supplementary material for: Bilateral vestibulopathy – insight in impact on quality of life and economic burden
Source: Eur Arch Otorhinolaryngol. 2025 Oct 14;283(2):711–8. doi: 10.1007/s00405-025-09692-3 (PMC12987859; doi:10.1007/s00405-025-09692-3)
Supplement: Supplementary file 1 — Supplementary Material 1 (PDF 507 KB) [file 405_2025_9692_MOESM1_ESM.pdf]

Supplementary materials

1. Patients survey

Since we would like to get an understanding of what your condition has cost you so far, we have prepared a few questions for you. These questions will also be reviewed with you on the day of the examination.

1. How often did you visit your GP in the last year concerning BV related complaints?

2. Did you visit a neurologist? ◯ Yes ◯ No
   a. In which hospitals?
   b. How often since the onset of your complaints?
   c. How often in the past year?

3. Did you visit a otorhinolaryngologist? ◯ Yes ◯ No
   a. In which hospitals?
   b. How often since the onset of your complaints?
   c. How often in the past year?

4. Did you visit a psychologist? ◯ Yes ◯ No
   a. How many different psychologists?
   b. How often since the onset of your complaints?
   c. How often in the past year?

5. Did you visit a psychiatrist? ◯ Yes ◯ No
   a. In which hospitals/clinics?
   b. How often since the onset of your complaints?
   c. How often in the past year?

6. Have you ever visited an alternative healthcare practitioner (for example: homeopathy, acupuncture, psychic healer, etc.)? ◯ Yes ◯ No
   a. Which one(s)?
   b. What were the costs?

7. What diagnostic tests have you undergone in relation to your symptoms, and how often since the onset of your symptoms?

**Diagnostic tests**	**Yes/No**	**How often in last year and total duration of disease?**
Blood	◯ Yes ◯ No	
CT: A scan in which a table passes through a donut-shaped scanner.	◯ Yes ◯ No	
MRI: A scan where you lie on a table that moves into a tunnel-like structure. You must remain very still while inside the tunnel.	◯ Yes ◯ No	
Vestibular test involving following dots and using a rotating chair.	◯ Yes ◯ No	
Calorics (vestibular test, where warm and cold water is introduced into your ears)	◯ Yes ◯ No	
Other		

8. Which treatments have you tried? How often, and what were the associated costs?

9. Have you had to make adjustments in your home? Which ones? Costs?

10. Do you need any medical devices to help you in daily life?

11. Have you hired help at home due to your symptoms? For example, a housekeeper, gardener, home care, etc.? How often, for how long, and costs?

12. Do you drive a car ◯ Yes ◯ No.
   a. Do you use a taxi service or other transportation? (If yes, how often and what distances?)
   b. Does a family member/caregiver take you to places? (If yes, how often and what distances?)

13. Have you fallen in the past year? ◯ Yes ◯ No.
   a. How often, and what medical care did you need?

2. Expert opinion survey

Before Surgery

1. Which vestibular tests would you recommend before surgery?

- Audiogram	
- Torsion swing	
- Velocity step	
- Calorics	
- vHIT 3D	
- DVA	
- C-VEMP	
- O-VEMP	
- Perception platform (research)	
- Gait analysis (CAREN, only available in Maastricht)(Research)	

Post-surgery trajectory (first year)

2. How long would you recommend to let the wound recover after surgery before the VCI/VI can be used?
   - Estimation between ….. and ….. days

3. Location of rehabilitation: hospital/rehabilitation clinic/day admittance
   - (Optional) explanation: ………………………………………………………………………………………
4. In case of an admittance, which duration:
   - Between ….. days/weeks/months and ….. days/weeks/months

5. Would you recommends expanding the use of the VCI/VI per day/week? **Yes/No**
   - In case yes:

- First week:		Hours a day
- Second week:		Hours a day
- Third week:		Hours a day
- Fourth week		Hours a day
- Fifth week		Hours a day
- Sixth week		Hours a day
- Comments:		

<u>Guidance</u>

6. *Audiologist*

- Starting with # amount of visits:		Times a day/week
- During which period of time		Days/weeks
- Tapering to		Visits per week/month/year
- Estimated time of follow-up		Days/weeks/months
- (optional) description		

7. *Technical team for VI*

- Starting with # amount of visits:		Times a day/week
- During which period of time		Days/weeks
- Tapering to		Visits per week/month/year
- Estimated time of follow-up		Days/weeks/months
(optional) description		

8. *Physical therapy*

- Starting with # amount of visits:		Times a day/week
- During which period of time		Days/weeks
- Tapering to		Visits per week/month/year
- Estimated time of follow-up		Days/weeks/months
(optional) description		

9. *Psychological guidance*

- Starting with # amount of visits:		Times a day/week
- During which period of time		Days/weeks
- Tapering to		Visits per week/month/year
- Estimated time of follow-up		Days/weeks/months
(optional) description		

10. *Social worker*

- Starting with # amount of visits:		Times a day/week
- During which period of time		Days/weeks
- Tapering to		Visits per week/month/year
- Estimated time of follow-up		Days/weeks/months
(optional) description		

<u>Vestibular examination</u>

11. Which vestibular tests would you recommend post-surgery and how often?

	Frequency	*At which moments (for example after 1 week, after .., etc)*
- Audiogram		
- Torsion swing		
- Velocity step		
- Caloric		
- vHIT 3D		
- DVA		
- C-VEMP		
- O-VEMP		
- Perception platform (research)		
- Gait analysis (CAREN, only available in Maastricht)(Research)		

12. How often should the patient visit the otorhinolaryngologist? …… times

- At which moments (for example 1 week after surgery and….)	

3. Frequency distribution of EQ-5D-5L and ICECAP-A

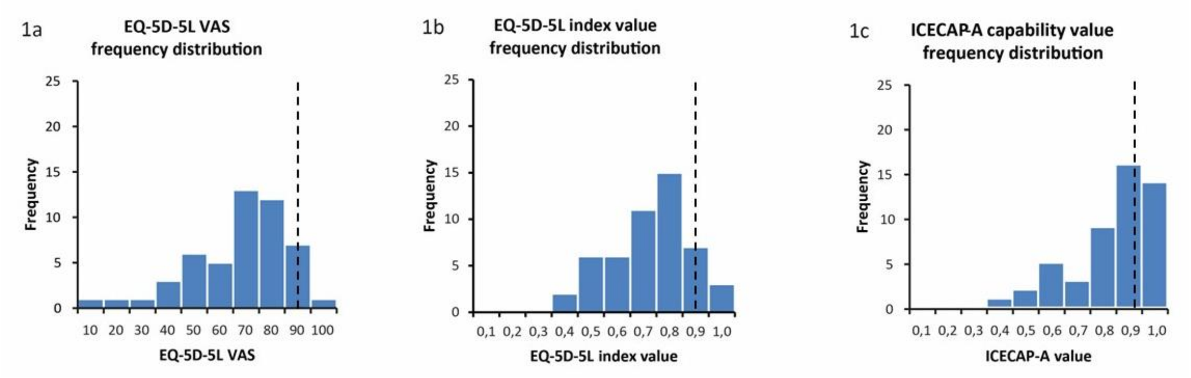

Figure a) *EQ-5D-5L VAS frequency distribution;* ***b)*** *EQ-5D-5L utility frequency distribution;* ***c)*** *ICECAP-A capability value frequency distribution. The dotted lines indicate the reference mean values based on the Dutch population tariff*

4. Total mean costs per cost item per patient, as calculated from 50 BV patients, divided into the last year of the disease and the total duration of disease period

			COSTS IN LAST YEAR OF DISEASE				COSTS IN TOTAL DISEASE PERIOD			
		Unit costs (€)	***n***	***Mean (€)***	***Min (€)***	***Max (€)***	***n***	***Mean (€)***	***Min (€)***	***Max (€)***
**HEALTH CARE COSTS**										
DOCTOR VISITS										
GP		28[A]	20	49				Not estimated		
OTORHINOLA-RYNGOLOGIST		109[A]	33	181	0	873	50	639	109	2618
NEUROLOGIST		109[A]	15	70	0	655	39	226	0	1091
EXAMINATIONS										
BLOOD		Individual[B]	0				15	26	0	194
IMAGING CT/MRI		595.42 [C]	1	12	0	595	43	1048	0	3573
VESTIBULAR TESTING	Vestibular testing incl rotary chair, calorics	[D]	21				50			
	vHIT	[D]	18				24			
	VEMP	[D]	0				3			
	Other	Individual	0				1			
	Total		21	344	0	842	50	1534	676	4034
AUDIOGRAM		96.84[C]	21	38	0	97	50	232	97	1065
TREATMENT										
PSYCHOLOGICAL SUPPORT	General practice mental health worker	19 [A]	1	9	0	455	1	9	0	474
	Psychologist	90 [A]	4	11	0	179	10	55	0	55
	Psychiatrist	127 [A]	2	17	0	573	3	34	0	891
PHYSICAL THERAPY		35.35[A]					16	1249	0	33088
REHABILITATION		3154[A,E]					5	259	0	3155
BETAHISTINE		€2.40/ month [F]					6	7	0	66
OTHER		Various [G]					7	356	0	14400
ALTERNATIVE MEDICINE [1]		Various [G,H]					11	436	0	9000
MEDICAL DEVICES [2]		Various [G,H,I]					25	409	0	6742
HEALTH CARE COSTS DUE TO FALLS [3]		Individual [c]	6	248	0	12086				
**PATIENT AND FAMILY COSTS**										
CHANGES AT HOME[4]		Various [G]					13	153	0	4000
HELP AT HOME [5]		21-52/h [A,G]	8	1157	0	15436	8	4807	0	58683
TRAVEL EXPENSES	Total						29	7428	0	99840
	Taxi	€3.36 + €2.47/km [A]	2	59	0	1671				

	Public transport	€0.21/km [A]	5	58	0	1814				
	Omnibuzz	€0.21/km [A]	1	15	0	756				
	Family/ friends	€0.26/km [A]	27	595	0	9984				
**PRODUCTIVITY LOSSES**										
LOSS OF PRODUCTICITY AT PAID LABOR		€36.25/h [J,K]	7	2522	0	101268	15	11926	0	101268
TOTAL SOCIETAL COSTS				***5388 (95% CI 2407 – 10730)***				***30708 (95% CI23176 - 38914)***		
**AVERAGE PER ANNUM OVER THE REPORTED DISEASE DURATION**								***8863 (95% CI 5614 - 12785)***		

N is the number of patients which consumed these costs, the mean costs is the mean amount per 50 patients. vHIT = video Head Impulse Test. VEMP = Vestibular Evoked Myogenic Potentials.

[A] ECONOMIC HEALTH EVALUATION GUIDELINES 2024 <SPAN STYLE="BASELINE">[21]</SPAN>*; [B] CENTRAL DIAGNOSTIC LABORATORY; [C] PATIENT PRICE LIST MAASTRICHT UNIVERSITY MEDICAL CENTER+ 2021; [D] CONFIDENTIAL, BECAUSE IN THE DUTCH HEALTH-CARE SYSTEM, ORGANIZATIONS NEGOTIATE UNIT COSTS OF (SOME OF) THEIR PRODUCTS WITH HEALTH-CARE INSURANCE COMPANIES; [E] TEN HOURS OF REHABILITATION THERAPY; [F] PHARMACOTHERAPEUTIC COMPASS; [G] COST SURVEY; [H] AVERAGE OF THE FIRST TEN PRICES GOOGLE SEARCH 24 NOVEMBER 2021; [I] DUTCH DATABASE OF MEDICINE AND MEDICAL DEVICES (GIP); [J] PRODISC; [K] FRICTION METHOD OF ECONOMIC HEALTH EVALUATION GUIDELINES 2024.*

[1] ALTERNATIVE MEDICINE INCLUDED ACUPUNCTURE, HOMEOPATHY, OSTEOPATH, ALTERNATIVE MEDICINE PRACTINIONER, MAGNETIZER, NATUROPATHIC DOCTOR, PSYCHIC, REIKI, HEALING COACH, CHIROPRACTOR, BIOPHYSICAL POSITION CORRECTION. [2] MEDICAL DEVICES: PRISM GOGGLES, ROLLING WALKER WALKING STICK, WHEELCHAIR, ELECTRIC BIKE, TRICYLE, SCOOTER, NORDIC WALKING STICKS, REAR VIEW MIRROR ON BICYCLE, FLASHLIGHT, SHOPPING TROLLEY, SUNGLASSES. [3] 17 PATIENTS REPORTED REGULAR FALLS, HOWEVER ONLY 6 HAD HEALTH CARE COSTS THE LAST YEAR DUE TO THE FALLS. [4]. EXTRA BANISTER, HANDLES, SHOWER SEAT, MULTIPLE BRIGHT SPOTS/LIGHT SENSORS, RAMPS, STAIRLIFT, RAISED TOILET SEAT, MICROCAR. [5] CLEANING LADY, GARDNER, HOME CARE, PAINTER

5. Hypothetical patient trajectory for vestibulocochlear implantation (first year), as defined by a group of 10 experts in vestibular medicine

	Opinions of:	*Test or specialist*	*Unit costs (€)*	*Mean nr of tests/ visits*	*Range nr of tests/visits*	*€*
**PRE-IMPLANTATION**						
**VISITS**		identical to CI trajectory				included in CI trajectory
**EXAMINATIONS**	2 otorhinolaryngologist, 5 audiologists	Torsion swing + caloric test	A	0*	0 – 1	
		velocity step	A	1	0 – 1	
		Only caloric	A	0	0 – 1	
		3-Dimensional Video Head Impulse Testing	A	1	0 – 1	
		Dynamic Visual Acuity	A	1	0 – 1	
		Cervical and ocular Vestibular Evoked Myogenic Potentials	A	1	0 – 1	
		Perception platform	Research B	0	0 – 1	
		Gait analysis	Research B	0	0 – 1	
SUBTOTAL PRE-IMPLANTATION EXAMINATIONS						**985**
**IMPLANTATION PROCEDURE**		Included in CI trajectory				
**POST-IMPLANTATION FIRST YEAR**						
VISITS	1 otorhinolaryngologist, 1 psychologist	Vestibular implant technical support	37 C	9	5 – 12	
	2 otorhinolaryngologists, 5 audiologists	Otorhinolaryngologist (academic)	109 C	1*	(1 – 5)	
	1 otorhinolaryngologist, 4 audiologists, 1 psychologist	Physical therapist	35 C	19	(5 – 48)	
	1 otorhinolaryngologist, 4 audiologists, 1 psychologist	Psychologist	90 C	5	(0 – 12)	
	1 otorhinolaryngologist, 1 audiologist, 1 social worker	Social worker	115 C	0	(0 – 0)	
SUBTOTAL VISITS						**1564**
EXAMINATIONS	2 otorhinolaryngologists, 5 audiologists	Torsion swing + caloric	A	2*	(1 – 4)	
	2 otorhinolaryngologists, 5 audiologists	Only torsion swing	A	0	(0 – 3)	
	2 otorhinolaryngologists, 5 audiologists	Velocity step	A	2	(0 – 4)	
	2 otorhinolaryngologists, 5 audiologists	3-Dimensional Video Head Impulse Testing	A	4	(1 – 12)	
	2 otorhinolaryngologists, 5 audiologists	Dynamic Visual Acuity	A	4	(0 – 12)	
	2 otorhinolaryngologists, 5 audiologists	Cervical and ocular Vestibular Evoked Myogenic Potentials	A	2	(0 – 3)	
	2 otorhinolaryngologists, 5 audiologists	Perception platform	A	3	(0 – 12)	
	2 otorhinolaryngologists, 5 audiologists	Gait analysis	A	3	(0 – 12)	
SUBTOTAL POST-IMPLANTATION EXAMINATIONS						**3987**
SUBTOTAL VESTIBULAR						**6536**
**COCHLEAR IMPLANT TREATMENT TRAJECTORY WITHOUT IMPLANT AND PROCESSOR A, D**						**26070**
**TOTAL CLINICAL COSTS OF VESTIBULOCOCHLEAR IMPLANTATION TREATMENT TRAJECTORY FIRST YEAR WITHOUT IMPLANT AND PROCESSOR**						**32605**

[A] CONFIDENTIAL; [B] COSTS NOT YET KNOWN; [C] HEALTH ECONOMIC EVALUATION GUIDELINES 2024; [D] PATIENT PRICE LIST MAASTRICHT UNIVERSITY MEDICAL CENTER+ 2021. THE COCHLEAR IMPLANT TREATMENT TRAJECTORY (MAASTRICHT UMC+) INCLUDED: PRE-IMPLANTATION: ONE AUDIOGRAM, TWO VISITS TO AN AUDIOLOGIST, TWO VISITS TO THE COCHLEAR IMPLANT COORDINATOR, TWO VISITS TO AN OTORHINOLARYNGOLOGIST, A CT AND/OR MRI, A VISIT TO A SOCIAL WORKER/PSYCHOLOGIST, AND A CALORIC TEST. POST-IMPLANTATION: FOUR AUDIOGRAMS, SEVEN FITTING SESSIONS, FOUR MEETINGS WITH A SPEECH THERAPIST, ONE VISIT TO A SOCIAL WORKER, TWO VISITS TO AN OTORHINOLARYNGOLOGIST, AND A CALORIC TEST AFTER 9 MONTHS.

The vestibular treatment trajectory was divided into the following components: pre-implantation vestibular tests, pre-implantation visits, implant procedure, post-implantation visits, and post-implantation tests. Per component, the average amount of units was calculated recommended by the experts. The sum of the mean reported additional care costs and the costs of the routine cochlear implant treatment trajectory (without the implant and processor) was presented as an approximation of tota
